# Detection of subtype-specific breast cancer surface protein biomarkers via a novel transcriptomics approach

**DOI:** 10.1042/BSR20212218

**Published:** 2021-12-07

**Authors:** Daniele Mercatelli, Francesco Formaggio, Marco Caprini, Andrew Holding, Federico M. Giorgi

**Affiliations:** 1 Department of Pharmacy and Biotechnology, University of Bologna, 40126 Bologna, Italy; 2 York Biomedical Research Institute, University of York, Heslington, York, YO10 5DD, U.K.

**Keywords:** bioinformatics, biomarkers, breast cancer, master regulators, surfaceome, transcriptomics

## Abstract

Background: Cell-surface proteins have been widely used as diagnostic and prognostic markers in cancer research and as targets for the development of anticancer agents. So far, very few attempts have been made to characterize the surfaceome of patients with breast cancer, particularly in relation with the current molecular breast cancer (BRCA) classification. In this view, we developed a new computational method to infer cell-surface protein activities from transcriptomics data, termed ‘SURFACER’.

Methods: Gene expression data from GTEx were used to build a normal breast network model as input to infer differential cell-surface proteins activity in BRCA tissue samples retrieved from TCGA versus normal samples. Data were stratified according to the PAM50 transcriptional subtypes (Luminal A, Luminal B, HER2 and Basal), while unsupervised clustering techniques were applied to define BRCA subtypes according to cell-surface proteins activity.

Results: Our approach led to the identification of 213 PAM50 subtypes-specific deregulated surface genes and the definition of five BRCA subtypes, whose prognostic value was assessed by survival analysis, identifying a cell-surface activity configuration at increased risk. The value of the SURFACER method in BRCA genotyping was tested by evaluating the performance of 11 different machine learning classification algorithms.

Conclusions: BRCA patients can be stratified into five surface activity-specific groups having the potential to identify subtype-specific actionable targets to design tailored targeted therapies or for diagnostic purposes. SURFACER-defined subtypes show also a prognostic value, identifying surface-activity profiles at higher risk.

## Introduction

While advances in therapeutic options and diagnostic tools have significantly improved breast cancer (BRCA) survival, BRCA incidence has continuously increased over the years, especially in high-income countries [1,2]. It is expected that almost 3.2 million women will be diagnosed with BRCA by 2040, counting about 1 million new deaths [3]. Clinically, the classification of BRCA relies on histopathological appearance and expression of hormone and growth factors receptors. Transcriptome profiling of BRCA patients based on the expression levels of 50 selected genes (PAM50) led to the definition of five clinically relevant BRCA intrinsic molecular subtypes, namely Luminal A (LumA), Luminal B (LumB), HER2-enriched (HER2+), Basal (Basal) and Normal-like (Normal) [4–7]. However, the PAM50 intrinsic subtype classification did not identify claudin-low tumors [7]. Identifying new cancer subtype-specific biomarkers will provide a more rapid diagnosis and finely tuned classification of BRCA patients to improve disease management.

For decades, surface antigens have been frequently adopted for diagnostic and classification purposes in oncology. Surface antigens are usually optimal biomarker candidates because they are accessible for both antibody-based diagnostic and pharmacological interventions [8]. Surface antigens can also be frequently detected from blood samples for diagnosis and to monitor response to therapy, for example, CA 15-3 or carcinoembryonic antigen (CEA) [9]. Testing expression levels of HER2 has been a well-established method to identify endocrine-sensitive BRCA patients or to select patients that would benefit from anti-HER2 therapy, respectively [10]. Human proteins exposed on the cell-surface represent an important determinant of the complex interface regulating interactions between the intracellular and the extracellular space, and collectively form the surface proteome or ‘surfaceome’ [11]. Surface proteins are ideal candidate nodes in cellular networks due to four properties: (1) accessibility: they can be targeted by drugs and molecular detectors without crossing the plasma membrane [12], (2) pleiotropicity: every human cell possesses surface proteins [11], (3) precedence: surface receptors often act as the trigger of signaling cascades [13] and (4) specificity: cell surface markers are frequently associated with specific cell lineages and differentiation states, both in normal development and diseases such as cancer [14]. Therefore, a focused analysis on cell-surface protein expression and relationships has the potential to improve molecular characterization of distinct BRCA subtypes to sustain disease management. For example, it has been recently demonstrated that expression of genes encoding for cell-surface proteins can distinguish between two different molecular and histologic prostate cancer subtypes, prostate adenocarcinoma and neuroendocrine prostate cancer [15]. While mass spectrometry-based proteomics techniques are far from being implemented in routine clinical settings, RNA-seq has successfully entered clinical diagnostic protocols as demonstrated with the PAM50 classification [5,6]. However, individual gene expression profiles are often not sufficient at predicting actual surface protein abundance [16]. Therefore, we have developed a novel method, called ‘SURFace marker Assessment from Combined Expression analysis in R’ (SURFACER), to infer surface protein abundance from RNA-Seq data on a sample-by-sample basis. SURFACER is a direct extension of experimentally validated algorithms to accurately predict protein activity in cancer using weighted aggregation of gene expression profiles [17,18]. The SURFACER method relies on the interrogation of a context-specific surface protein network to successfully predict driver genes (also known as ‘Master Regulators’) explaining the observed phenotype [17,19]. This approach, usually applied to the identification of transcription factors driving specific disease states, has been extended to identify surface proteins acting as master regulators. Thus, SURFACER can define transcriptional modules linked to a subset of surface proteins expression that can be furtherly used as models to stratify BRCA subtypes on the basis of cell-surface protein expression patterns. This approach is an extension of our previously validated method to infer surface protein activity accurately in response to plant toxins in leukemia cells [20].

The aim of the present study is to analyze and integrate clinical and transcriptomic data of BRCA tumor samples from The Cancer Genome Atlas (TCGA) database [6], to identify clinically relevant BRCA surface-specific subtypes and to validate our findings on tumor samples from an independent cohort, the Molecular Taxonomy of Breast Cancer International Consortium (METABRIC) database [21]. Relationships between surface-specific BRCA subtypes and PAM50 molecular subtypes will be highlighted.

## Materials and methods

### Datasets information

Three independent datasets were used in the present study: the TCGA breast cancer dataset, the GTEx normal breast dataset and the METABRIC breast cancer dataset. Data from both the TCGA and GTEx cohorts were download following the recount pipeline, which is designed to remove batch effects present in the two large human datasets [22]. First, we analyzed 909 cancer samples from 909 patients from the TCGA BRCA cohort, including only patients with survival clinical information, from the Firehose web portal (https://gdac.broadinstitute.org/). In order to have a consistent normal tissue reference of similar size, we extracted transcriptional data from 212 healthy breast samples from the GTEx database (https://gtexportal.org/home/datasets). As a second breast cancer dataset, we downloaded data from METABRIC, including 1980 primary breast cancer gene expression (microarray) samples, via the European Genome-phenome Archive (EGA) portal [21]. Associated clinical data were downloaded from cBioPortal (https://www.cbioportal.org/study/summary?id=brca_metabric) on 22 February 2021.

### SURFACER Pipeline

The full code to reproduce our analysis is given at the following GitHub URL: github.com/N0toriou5/SURFACER. All computational steps were performed on a Windows 10 Pro 64 bit workstation with a eight cores i7 processor (3.0 GhZ), 64 GB of RAM. Briefly, the SURFACER pipeline is divided into the following major steps:

- Network analysis to infer the context-specific surface proteins activity network.
- Cluster Analysis for SURFACER subtypes definition.
- Combined Differential Gene Expression/ Master Regulator Analysis to prioritize surface markers.

Below, a description of each major part of the pipeline.

### Coexpression-based normal breast surface activity network

We applied a recently developed coexpression-based gene network inference and interrogation algorithm, corto [18], to build a normal breast network model considering relationships between surface protein-encoding genes and the whole human transcriptome. Normal breast gene expression data were retrieved from GTEx breast tissue dataset (*n*=212). A list of 3088 unique identifiers corresponding to human surface-protein encoding genes was obtained by manual curation of a larger list obtained by intersection of the terms ‘cell surface’ (GO:0009986), ‘anchored component of external side of plasma membrane’ (GO:0031362), ‘apical plasma membrane’ (GO:0016324), ‘external side of plasma membrane’ (GO:0009897), ‘extrinsic component of external side of plasma membrane’ (GO:0031232), ‘intrinsic component of external side of plasma membrane’ (GO:0031233), ‘plasma membrane protein complex’ (GO:0098797), ‘plasma membrane signaling receptor complex’ (GO:0098802), ‘plasma membrane region’ (GO:0098590) and the *in silico* human surfaceome [11]. This manually curated list of surface proteins, expressed as gene symbols and NCBI gene ids, is available as Supplementary File S1.

All surface proteins were used as potential hubs (‘centroids’) to run the corto function, in order to build surface protein-centered coexpression networks. We used the following arguments: nbootstraps = 1000, *P*=10^−10^, nthreads = 8. The network was used as input to infer differential surface protein activity in tumor samples versus normal samples by using the mra-Corto function included in the corto package with the following settings: regulon = the normal reference surface network built with the corto function corto, minsize = 15, nperm = 1000. The mra-Corto function performs Master Regulator Analysis (MRA), an algorithm aiming at inferring protein activity by interrogating a regulon with specific transcriptional signatures [17,18]. MRA can be performed between sample groups (e.g. cancer versus non-cancer) or on a sample-by-sample basis, providing protein activity relative to the median of the dataset [23]. A surface activity matrix calculating surface proteins activity on a single-patient basis was therefore obtained by running the mra-Corto algorithm on the Variance-Stabilizing-Transformed (VST) [24] TCGA-BRCA expression matrix. For every surface protein subnetwork, genes are ranked according to the maximums of their correlation scores with the network centroid (i.e. the surface gene of interest), then the mra-Corto function calculates an enrichment score to reflect the enrichment of a given surface gene’s targets toward the top of the corresponding gene ranking generated, that is a positive value if target genes are up-regulated in the dataset, or a negative value if target genes are down-regulated. Then, a normalized enrichment score (NES) is obtained for a particular subnetwork, considering the size of the subnetwork itself.

### Differential gene expression analysis

We used EdgeR Bioconductor Package [25] to perform differential gene expression analysis on raw counts data tumor versus normal samples. PAM50 subtype classification was performed through the pamr package [26]. For differential expression, false discovery rate (FDR) values were obtained by Benjamini and Hochberg method [27]. If not differently stated, differentially expressed genes (DEG) where defined as showing a |log2FC| > 1 and an FDR ≤ 0.05. To define critical surface markers, DEGs were filtered for genes showing an absolute NES > 2 (FDR ≤ 0.05) calculated by MRA tumor versus healthy tissue (i.e. TCGA versus GTEx). Enrichment analysis was performed on Enrichr web server [28]. Limma Bioconductor package [29] was used to perform differential expression analysis on gene expression data from the METABRIC cohort versus normal samples from the GTEx cohort.

### Heatmap construction and cluster analysis

VST expression matrices of 909 patients from the TCGA cohort were used to calculate a single-patient surface protein activity heatmap through the mra-Corto function as described above. The rows (proteins) and columns (samples) were then ordered based on a hierarchical cluster by applying Ward’s method [30] with average linkage and Pearson’s correlation distance. According to elbow method, which suggested an optimal number of clusters between 4 and 6 (Supplementary Figure S1), patients were grouped into five clusters, renamed for similarity with PAM50 classification as follows: Basal-like, Lum1, Lum2, Lum3 and Mixed.

### PAM50 and SURFACER subtype classifications

The expression levels of the PAM50 panel genes from each of the 909 tumor samples from TCGA were used to carry out the intrinsic subtype classification of tumors by using the pamr R package. SURFACER network transcriptional models were generated, and assignment of METABRIC patients to the most likely SURFACER subtypes was obtained by Pearson correlation following the nearest shrunken centroids classifier method [26].

### SURFACER genotyping validation

To explore the accuracy of SURFACER in BRCA patient samples genotyping, TCGA dataset was divided into a training and a validation set (80–20%, respectively). The performance and accuracy of the following classification algorithms from the caret R package (v 6.0-90) [31] were assessed using 10-fold Cross-Validation: Support Vector Machines with Radial Basis Function Kernel (svm), Stochastic Gradient Boosting (gbm), Random Forest (rf), k-Nearest Neighbors (knn), Nearest Shrunken Centroids (pam), Greedy Prototype Selection (protoclass), Multi-Layer Perceptron (mlp), Linear Discriminant Analysis (lda), Bayesian Generalized Linear Model (bgm), Stabilized Nearest Neighbor Classifier (snn) and Neural Networks with Feature Extraction (pcan). The performance of the different ML algorithms was tested using the following two parameters: accuracy and the Cohen’s Kappa statistics.

### Survival model

Survival analysis was performed using the survival and survminer R CRAN packages. The effect of each selected surface gene on survival was estimated using a univariate Cox proportional hazard model with the survival information of the 909 patients of the TCGA cohort. Kaplan–Meier curves for each group were generated, and the survival distributions were compared using Log-Rank test. The same approach was followed with METABRIC data.

## Results

### The surfaceome of PAM50 subtypes

**Figure 1:**
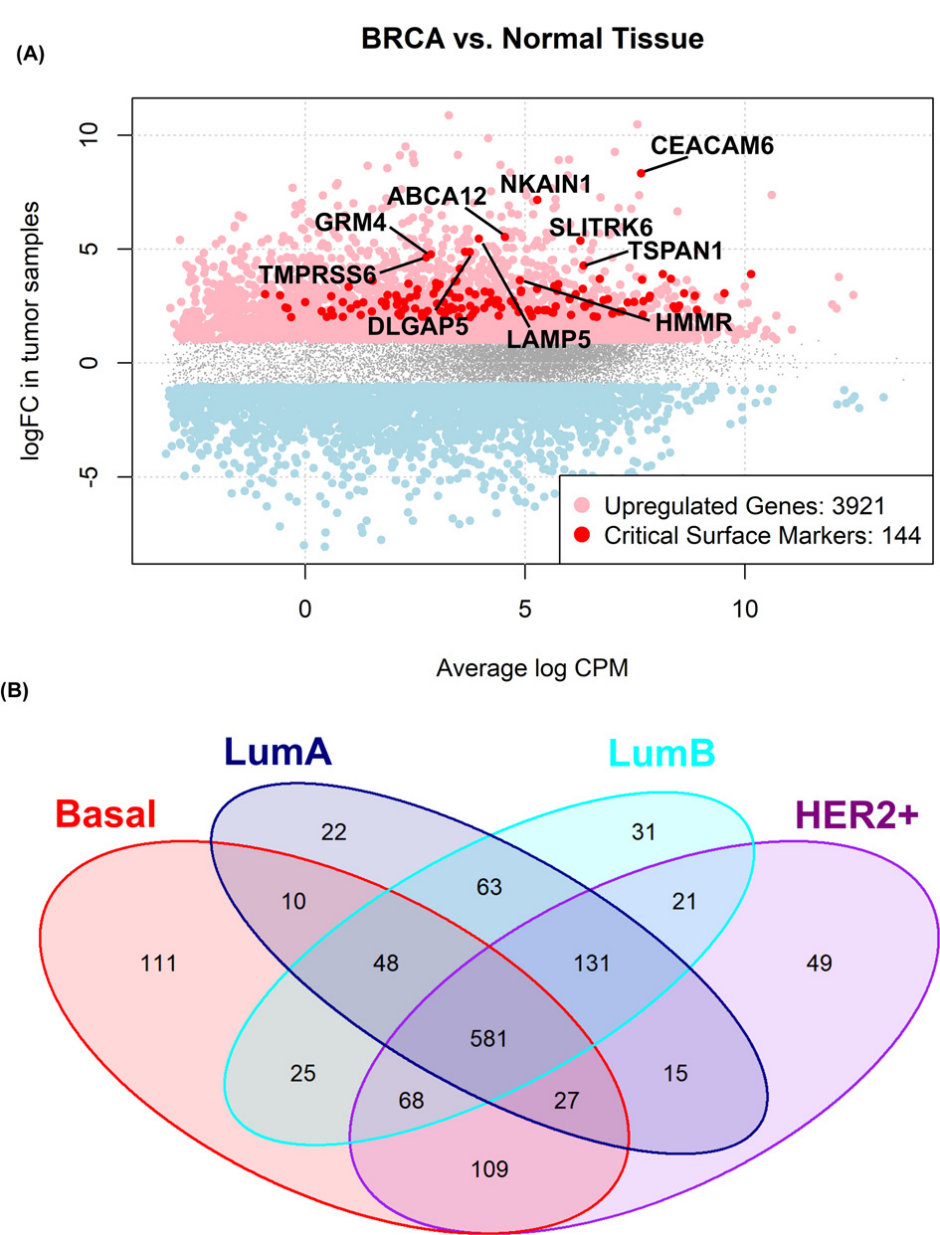
Up-regulated surface genes in BRCA (**A**) EdgeR logFC versus average log Counts Per Million (CPM) plot. In red, critically up-regulated surface proteins are highlighted. Top 10 critically enhanced BRCA surface markers are labeled. (**B**) Venn diagram showing the intersection between significant critically altered surface proteins in PAM50 intrinsic subtypes versus normal reference tissue from GTEx.

Batch-corrected raw counts data from TCGA-BRCA and GTEx breast tissue patients were merged together in a single raw gene expression counts matrix. In total, 954 tumor samples from the TCGA-BRCA dataset and 212 normal breast tissue reference counts from GTEx were kept in our analysis. Forty-five patients from TCGA lacking overall survival status information were discarded, leaving a total of 909 BRCA patients expression data. Single patients were assigned to histological groups using PAM50 gene markers [4,6,26] and differential expression analysis BRCA–TCGA versus normal breast tissue reference was performed on the whole cohort and on a subtype-specific basis. The complete transcriptome wide differential expression analysis is available in Supplementary Table S1 for the global breast cancer (TCGA) versus normal breast (GTEX) contrast, and for individual breast cancer subtypes versus normal breast in Supplementary Table S2 (PAM50 subtypes) and Supplementary Table S3 (SURFACER subtypes). Differential expression analysis results were furtherly filtered to focus on surface protein coding genes; this was attained by filtering differential expression results for the 3088 surface proteins included in the SURFACER curated list (Supplementary File S1). A normalized enrichment score for each surface protein was obtained by applying the mra-Corto algorithm, as explained in Materials and Methods. A total of 32 surface genes were found to be critically altered in BRCA versus normal tissue, 144 of which showed up-regulation at both mRNA expression, and inferred protein activity level (Figure 1A).

We then investigated differential activity of surface proteins for each intrinsic subtype versus normal reference. A total of 581 surface coding genes are differentially expressed in all PAM50 intrinsic subtypes versus normal reference tissue expression, while a subset of surface genes shows restricted differential expression characterizing each subtype: 111 genes are differentially expressed in basal patients only, 22 in luminal A, 31 genes in luminal B and 49 in HER2+ patients (Figure 1B). The surface protein HER2 (encoded by the ERBB2 gene) is in fact up-regulated in all subtypes when compared with normal breast tissue, however showing great differences in fold changes. In fact, while a log2FC = 5.5 can be detected in HER2+ patients versus normal reference, 2.2 and 3.4 log2FC characterized LumA and LumB samples, respectively. Basal samples showed the smallest difference, with an up-regulation of 1.5 versus normal samples. Differential expression values of all significant surface targets for each intrinsic subtype are given in Supplementary Table S1.

**Figure 2:**
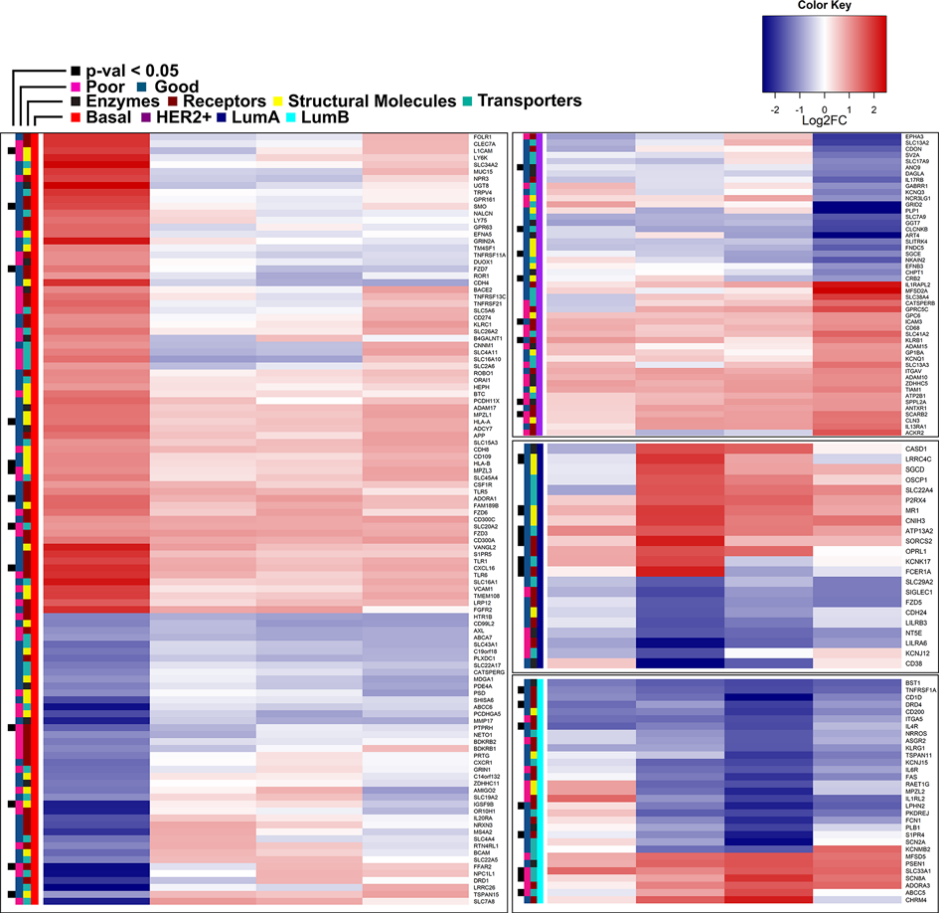
Heatmap of 213 PAM50 subtype-specific significant surface markers Association of surface markers with prognosis was evaluated by univariate Cox’s regression model coefficients, with positives coefficients indicating a poor prognosis (pink side bars), and negative coefficients indicating good prognosis (green-blue side bars). A black box corresponding to FDR value for Cox’s coefficients < 0.05 was indicated.

The 213 PAM50 subtype-specific surface genes are showed in Figure 2. As previously reported [32], up-regulation of Folate Receptor alpha (FOLR1) is characterizing the basal subtype, together with programmed death ligand 1 (PD-L1, CD274) [33], while the discovery of many other targets that are readily suitable for immunotargeted therapy can be enhanced by the SURFACER approach. Among these genes, a total of 35 differentially expressed surface genes were found to be significantly associated with overall survival in BRCA patients. Thirteen genes out of the 111 genes characterizing the basal subtype-restricted surface signature showed a prognostic value: an increased risk is expected for patients expressing high levels of FFAR2, IGSF9B, L1CAM, MPZL3, PTPRH and SLC20A2 (sodium-dependent phosphate transporter 2, PiT-2), while a protective effect was observed for ADORA1, CXCL16, FZD7, HLA-A, HLA-B, SMO and TSPAN15. Among the 8 genes mostly characterizing the HER2+ subtype, an increased risk was detected for SCARB2 and SPPL2A only, while the remaining (ANO9, CLCNKB, SGCE, CRB2, ICAM3 and KLRB1) were associated with an increased risk when down-regulated. Luminal subtypes showed a total of 14 genes significantly associated with overall survival, 6 in subtype A and 8 in subtype B. Increased risk was detected for ABCC5, SCN8A (another sodium channel) and SLC33A1. Indeed, sodium homeostasis is frequently altered in cancer, possibly due to the misexpression of key sodium channels and transporters like those identified by our study [34]. Down-regulation is associated with an increased risk for ATP13A2, CD1D, FCER1A, IL4R, KCNK17, LPHN2, LRRC4, MR1, SIP4R, SORCS and TNFRSF1. Surface genes were annotated to four functional classes, namely enzymes, receptors, transporters and structural molecules, and color labeled accordingly in Figure 2.

### TCGA breast cancer patients clustering according to surface protein activity

**Figure 3:**
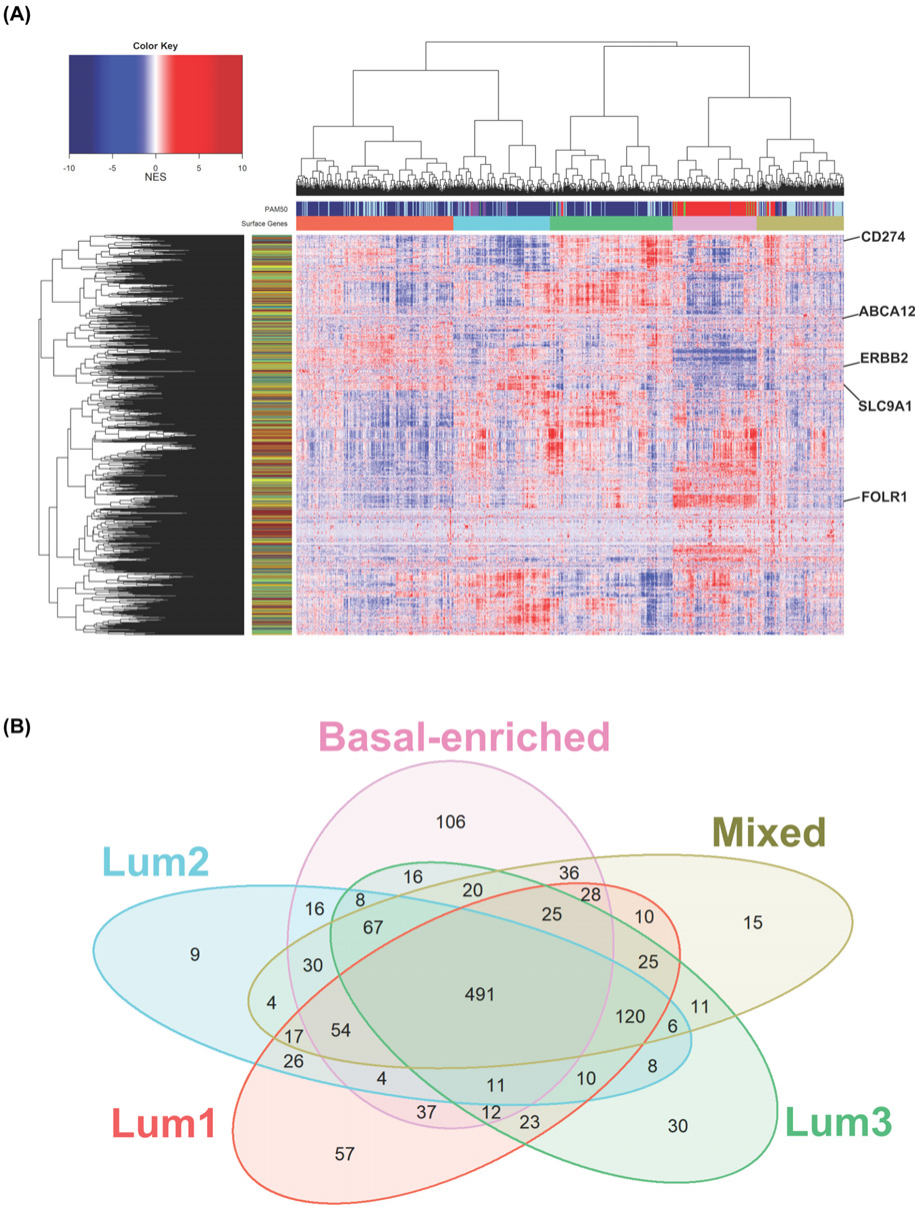
BRCA patients subtyping according to surface protein network activity (**A**) Surface protein network activity heatmap of the TCGA BRCA cohort. Patients were clustered together for surface proteins inferred activity, and relationships with PAM50 classification were indicated by PAM50 side bar (column). Rowside colors were added to label surface markers for functional class: enzymes (dark gray), receptors (dark red), structural molecules (yellow) and transporters (green). Few key surface markers are highlighted. (**B**) Venn diagram showing the intersection between significant critically altered surface proteins in SURFACER clusters versus normal reference tissue from GTEx.

The mra-Corto algorithm was run in sample-by-sample mode to obtain a single-patient surface activity score for each surface protein coding gene in the SURFACER list. This way, we obtained a dataset-wide readout of normalized surface activity scores across patients that we used to group patients into clusters showing similar patterns of surface protein activities: sample-to-sample distance was determined as 1-Pearson correlation coefficient, and hierarchical clustering was performed by Ward’s method [30]. Elbow method suggested 5 as the optimal number of clusters to subdivide TCGA patients according to surface proteins inferred activity (Supplementary Figure S1). Relationships between SURFACER subtypes and PAM50 classification are shown in Figure 3A: TCGA patients belonging to the basal PAM50 intrinsic subtype (red bar) were similarly clustered to the SURFACER subtype indicated in light pink, and therefore named ‘basal-like’. A small cluster mainly composed by PAM50 HER2+ and luminal samples, therefore renamed as ‘mixed’ subtype, was labelled by a khaki green bar. PAM50 Luminal A and B patients were represented by three different SURFACER clusters that we named as Lum1-3. To note, most of the TCGA cohort was assigned to the Luminal A subtype that may introduce a potential source of bias in the fine classification of luminal breast cancers according to surface proteins expression data. However, the current PAM50 classification is considered by several authors to be insufficient to fully recapitulate BRCA complexity. In fact, the PAM50 panel and intrinsic subtypes gene signature prototypes were obtained from bulk tissue data, and this can introduce a bias due to sampling procedures, as also discussed in [35]. Furthermore, one has to take into account that currently available large expression sets are poorly able to reflect BRCA at the population level [36]; thus, larger curated datasets will be required to refine predictions. In Supplementary Figure S2, the relative abundance of each SURFACER subtype into classic PAM50-assigned patients clusters is showed. To note, while few patients would be assigned to the normal-like subtype by PAM50 classification, our method identifies those samples as mostly basal-enriched samples. The advantage of SURFACER subtypes classification is, however, the possibility to identify subtype-specific actionable markers on the basis of protein activity at network level. Other BRCA classifications do exist, such as [37], but none is specifically surfaceome-oriented.

### Identification of the top markers of SURFACER subtypes

**Figure 4:**
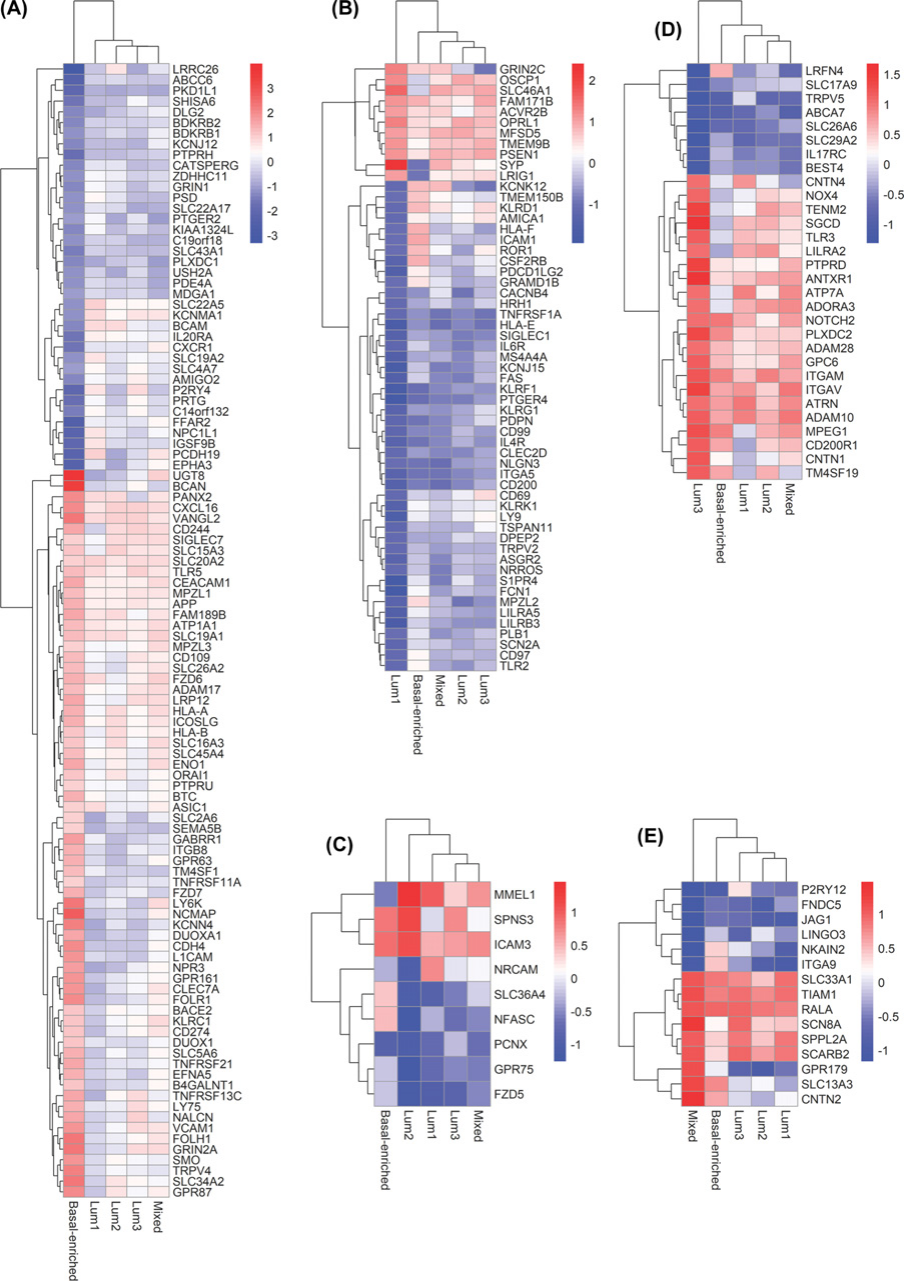
SURFACER subtypes-specific altered genes Heatmaps showing genes whose activity was differentially altered only in (**A**) Basal-enriched, (**B–D**) Lum1-3 and (**E**) mixed SURFACER subtypes.

The surfaceome of the five SURFACER clusters was then investigated following the same approach used to characterize PAM50 subtypes-specific surfaceomes. Raw differential expression tables containing the differential expression analysis versus normal reference for each SURFACER subtype is given in Supplementary Table S1, while a selection of all significant surface markers is given in Supplementary Table S2. Venn diagram showed that 491 surface protein coding genes are commonly deregulated in all SURFACER subtypes when compared with normal reference tissue gene expression, while few genes specifically characterize each subtype (Figure 3B). A 106 proteins signature defines the basal-enriched subtype (Figure 4A). Many genes that were showed to characterize PAM50 basal subtype are included in this signature, like FOLR1, CD274, and all markers also showing a significant prognostic value, as discussed above. Enrichment analysis was performed by uploading up-regulated and down-regulated basal-enriched genes into Enricher web server separately and interrogating the WikiPathways gene sets. While no significant positive enrichment for any pathway was detected, a significant negative enrichment of the ACE Inhibitor Pathway (FDR = 0.006379) was observed in basal-enriched subtype-restricted down-regulated surface markers. The Lum1 subtype was mostly characterized by down-regulation of 45 surface markers. By uploading these 45 genes into Enrichr web server, a significant enrichment of the following WikiPathways was observed: Platelet-mediated interactions with vascular and circulating cells, Small Ligand GPCRs, Apoptosis Modulation by HSP70 (FDR = 0.021). The Lum2 subtype was characterized by a small signature of nine genes. Three genes (Membrane Metalloendopeptidase Like 1 [MMEL1], Sphingolipid Transporter 3 [SPNS3] and Intercellular Adhesion Molecule 3 [ICAM3]) were found to be significantly up-regulated in this subtype, which makes these genes ideal candidates for Lum2-specific targeting. Among these, ICAM3 up-regulation was previously shown to be correlated with tumor staging and to mediate tumor metastasis [38]. No relevant pathway enrichment was detected for the Lum2 subtype. Up-regulation of canonical and non-canonical Notch signaling (FDR = 0.020) was observed in Lum3, while a positive enrichment of RalA downstream regulated genes (FDR = 0.029) was observed in the mixed subtype.

### Validation of the SURFACER method in BRCA genotyping

**Figure 5:**
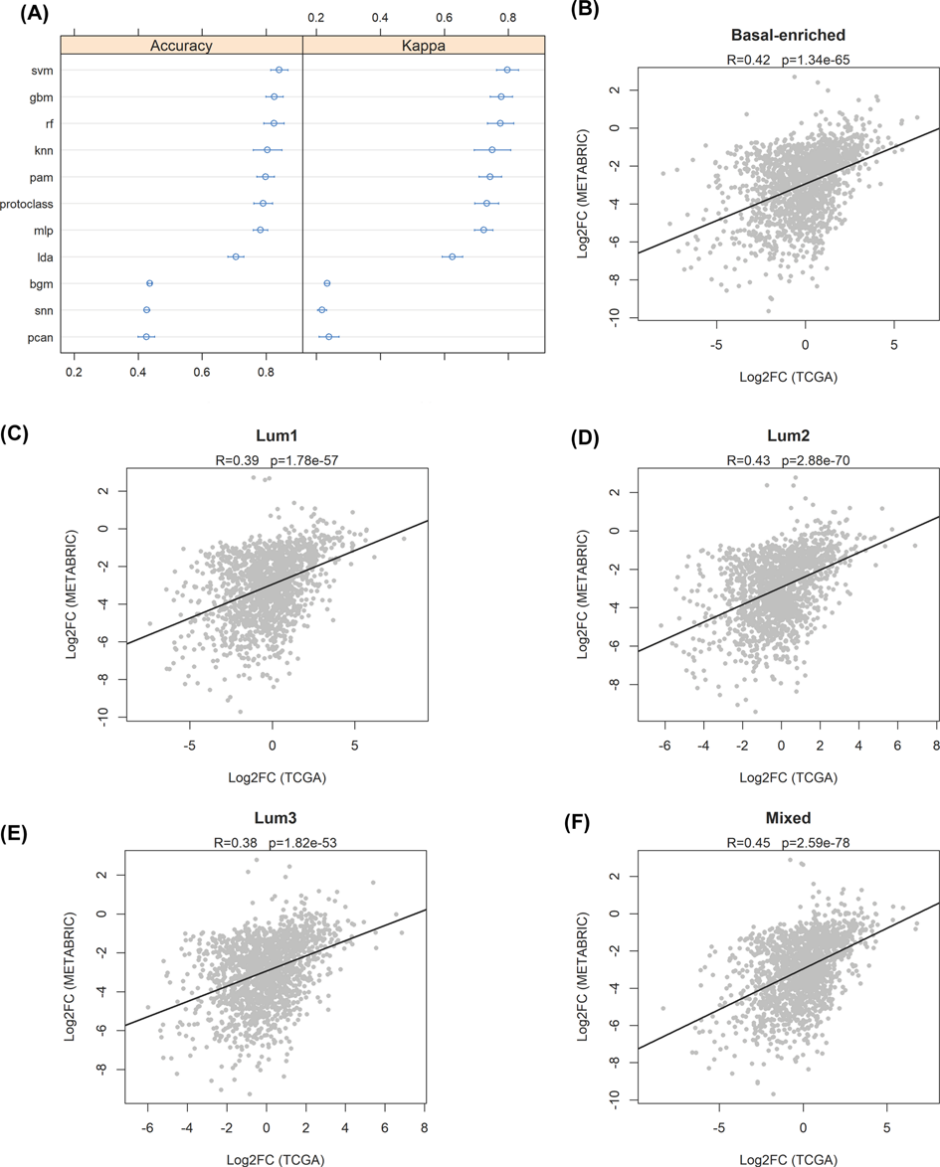
SURFACER genotyping validation (**A**) Estimation of the performance of several ML algorithm on sample classification. Accuracy and Cohen’s Kappa of the following ML algorithms were plotted (confidence level: 0.95): Support Vector Machines with Radial Basis Function Kernel (svm), Stochastic Gradient Boosting (gbm), Random Forest (rf), *k*-Nearest Neighbors (knn), Nearest Shrunken Centroids (pam), Greedy Prototype Selection (protoclass), Multi-Layer Perceptron (mlp), Linear Discriminant Analysis (lda), Bayesian Generalized Linear Model (bgm), Stabilized Nearest Neighbor Classifier (snn) and Neural Networks with Feature Extraction (pcan). (**B–D**) Scatterplots showing the Log2 fold changes of differential expression analysis in TCGA (*x*-axis) and METABRIC (*y*-axis) samples versus normal breast tissue reference samples. Pearson correlation coefficients (*R*) and significance of each analysis are reported.

The accuracy of SURFACER in breast cancer genotyping was tested by estimating the performance of 11 different machine learning (ML) algorithms on assigning patients to the correct SURFACER subtype. The 10-fold cross validation procedure was performed to test accuracy, sensitivity and specificity of ML predictions. The TCGA dataset was randomly splitted into two datasets, a training set and a validation set, with a 80–20% split. Thus, the 909 samples in the TCGA cohort were allocated as follows: 638 samples in the training set, 271 samples in the validation set. A very good balance in class representation was achieved between training and validation (Basal-enriched, 15.3–15.0%; Lum1, 28.7–28.9%; Lum2, 17.6–17.8%; Lum3, 22.5–22.2%; Mixed, 15.9–16.1%, respectively). Accuracy and Cohen’s Kappa were used to estimate the performance of both linear and non-linear ML classification algorithms. Figure 5A shows the results of the analysis: among top performers, Support Vector Machines with Radial Basis Function Kernel (svm), Stochastic Gradient Boosting (gbm) and Random Forest (rf) showed the highest accuracy (over 0.8), while *k*-Nearest Neighbors (knn), Nearest Shrunken Centroids (pam), Greedy Prototype Selection (protoclass) and Multi-Layer Perceptron (mlp) showed good performances, with accuracy values near 0.8. Worst performances were showed by Bayesian Generalized Linear Model (bgm), Stabilized Nearest Neighbor Classifier (snn) and Neural Networks with Feature Extraction (pcan) algorithms. The svm algorithm was found to be the best performer, showing an overall accuracy of 0.833 in predicting the correct class in the validation set (confidence interval = 0.777 < 0.833 < 0.8846, *P*<2.2e-16) and a Cohen’s Kappa = 0.788. The svm predictor showed a 99% accuracy in predicting the Basal-enriched class (Sensitivity = 1.00, Specificity = 0.99), a 86% accuracy in predicting the Lum1 class (Sensitivity = 0.79, Specificity = 0.92), a 87% accuracy in predicting the Lum2 class (Sensitivity = 0.78, Specificity = 0.97), a 91% accuracy in predicting the Lum3 class (Sensitivity = 0.90, Specificity = 0.93), and a 85% accuracy in predicting the Mixed class (Sensitivity = 0.72, Specificity = 0.97).

Since the svm algorithm was found to be the best predictor to classify BRCA patients according to SURFACER genotyping, we used this algorithm trained on the entire TCGA dataset to classify samples in the METABRIC cohort. Subsequently, a differential expression analysis comparing METABRIC samples to GTEx normal reference samples was performed using limma’s empirical bayes statistics, considering that no rawcounts data but only expression data for the METABRIC samples were available. We then compared the log2 fold changes characterizing the differential expression of surface genes of each SURFACER-defined subtype in METABRIC versus the TCGA analysis discussed above measuring the strength of association by Pearson’s correlation coefficient. A good significant correlation between the two analyses was detected in every contrast, as showed in Figure 5B–F.

As a proof of concept, we tested the activity of SLC9A1 (NHE1), a well-known Na^+^/H^+^ Exchanger known to be involved in breast cancer metastasis, especially in basal breast cancer subtypes (Supplementary File S2) [39,40] .

### The prognostic value of SURFACER subtypes

**Figure 6:**
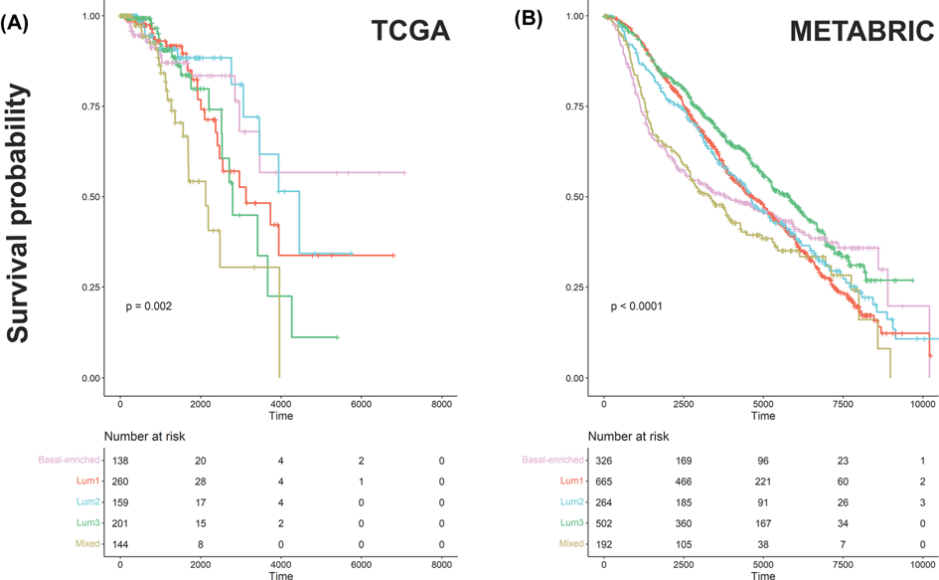
Survival analysis Overall survival of SURFACER clusters. Kaplan–Meier curves corresponding to the 5 SURFACER clusters were analyzed in both the (**A**) TCGA and (**B**) METABRIC cohorts. Statistical significance was evaluated by Log-Rank test.

To investigate clinical relevance of SURFACER clusters, we performed survival analysis taking the overall survival of TCGA patients as outcome variable (Figure 6A). Overall survival (OS) analysis at 5 years (∼2000 days) revealed that patients belonging to the mixed subtype experience significantly worse outcomes compared with the other subtypes. A significant worst prognosis at 11 years follow-up (∼4000 days) is showed by patients belonging to the mixed phenotype, while better outcomes are observed for basal-enriched patients. However, Kaplan–Meier curves beyond 2000 days are less reliable due to low number of events recorded during long-term follow-ups. To investigate clinical relevance of SURFACER subtypes on similarly large dataset, we obtained gene expression and clinical data from the METABRIC Consortium. SURFACER subtypes-specific surface activity signatures were defined by nearest shrunken centroids classifier method, and patients assigned to the most correlated surface activity subtype by Pearson correlation, as described in materials and methods. As showed in Figure 6B, at 5 years both mixed and basal-enriched subtypes show the worst prognosis, while at 11 years basal-enriched OS was significantly better compared with mixed subtype patients. Similar OS were observed for Lum1-3 at 5 years, while better outcomes are observed for Lum3 patients at 11 years. At longer follow-ups, both Lum3 and basal-enriched patients showed better prognosis, while mixed subtype patients were the ones showing worst outcomes.

Most of the differences showed between TCGA and METABRIC cohorts KM curves may be in part dependent to the difference in both sample composition and the completeness of associated clinical data. In fact, it was previously observed that molecular subtypes composition between TCGA and METABRIC datasets is variable, and it does not adequately capture BRCA real subtype distribution at the population level [36].

## Discussion

The availability of relevant biomarkers used for BRCA patients’ classification, diagnosis and prognosis, and therapeutic options have increased significantly over the last decade [41]. Recent advances on next-generation sequencing (NGS) techniques, together with the decrease of NGS associated costs, have tremendously expanded the use of RNA-seq in clinical practice, leading to the production of a large amount of expression datasets whose information has still only in part decoded.

BRCA molecular subtype classification, defined by PAM50, was originally proposed to add prognostic/predictive value to invasive BRCA molecular characterization. However, most of the relevant information about tumor intrinsic biology can be masked by bulk sequencing, and some specific properties for the PAM50 subtypes reflect changes in the patients’ tumor microenvironment (TME) instead of specific molecular changes occurring in cancer cells. Considering that BRCA can be seen as a disease shaped through the complex relationship between cancer cells and the local environment, and that this relationship can be partly investigated by focusing on cell surface proteins, which are the direct mediators of most of the exchanges occurring between cancer cells and the TME, we developed SURFACER, a bioinformatics approach to infer cell-surface protein activity from context-specific gene regulatory networks [18,42]. One of the major advantages of our approach is that it may infer protein abundance from gene expression data, by overcoming known limitations in predicting actual surface protein abundance by applying weighted aggregation of gene expression profiles and gene network analysis to rank all differential surface protein activities in tumor samples versus healthy tissues [17,18,42].

We showed that each PAM50 intrinsic subtype can be described by a specific surface protein activity signature, which includes both known surface markers (like e.g. FOLR1 up-regulation in the basal subtype [32]) and less characterized proteins that may be studied for subtype-specific diagnostic or therapeutic purposes (e.g. targeted therapy). Since no clear membrane protein markers have been described so far for LumA, LumB and TNBC subtypes, our novel approach opened the possibility to identify such markers also for these subtypes, raising the possibility to target them in a subtype-specific manner.

BRCA patients can be stratified into five surface activity-specific groups, showing some similarities with PAM50 intrinsic subtypes, but having the potential to identify subtype-specific actionable targets to design tailored targeted therapies, or for diagnostic purposes. SURFACER-defined subtypes show also a prognostic value, identifying surface-activity profiles at higher risk. The mixed phenotype, the one showing the worst prognosis, is characterized by the restricted deregulation of 15 genes at network level, including some motility/metastatic potential related genes, like G-protein RalA (RALA) [43], T-cell lymphoma invasion and metastasis-inducing protein (TIAM1) [39], and NaV 1.6 channels, encoded by the SCN8A gene [40], which may cooperate in shaping an aggressive behavior.

The identification of optimal surface markers for both diagnostic and therapeutic purposes is a known challenge in the development of clinically relevant cancer-targeting therapies [44]. While most of the quantitative proteomic techniques to identify protein markers are currently far from being implemented in routine clinical settings, expression data are increasingly available from large patients cohorts, along with clinical information. A previous attempt to investigate BRCA surfaceome was performed by da Cunha in [45], where the authors identified targets to be validated for diagnostic or therapeutic purposes. However, the advantage of our approach is that it not only relies on gene expression profiles but also can predict surface protein abundance by inference from coexpression network data, thus identifying potential targets having a functional relevance in the specific disease. Here, we showed that our network activity approach may identify functional alterations whereas the only differential expression information is not sufficient to identify relevant cell-surface markers, and that BRCA genotyping according to the surfaceome can be achived by our pipeline with a good reproducibility. The availability of large-scale -omics datasets, in this case the TCGA and METABRIC breast cancer datasets, combined by the development of specific pipelines like SURFACER, is pivotal in identifying future cancer biomarkers.

Together with transcriptional regulators, surface proteins act as checkpoint modules [46] for tumor-sustaining signal transduction, and as obvious subjects for future molecular and translational cancer research. Their convenient accessibility makes them also ideal biomarkers and biotargets for diagnostic tests and personalized therapeutical strategies, both for traditional pharmacology [47] and for T-cell-mediated immunotherapy [48].

By analyzing patients’ transcriptomes and associated clinical data, SURFACER is able to predict tissue-specific cell-surface markers showing altered activity in pathological states, also making it possible to stratify patients according to clinically relevant specific molecular subtypes. The SURFACER approach can be extended to every cancer type, and an integrated pan-cancer approach will help defining critical surface markers beyond canonical cancer type borders [49]. SURFACER is fully generalizable to other human pathologies as well, which may benefit from the characterization of specific biomarkers, such as autoimmune syndromes [50], genetic diseases [51] and virus-mediated neuroinflammation, where central nervous systems cells are presenting peculiar surfaceomes upon infection [52].

## Supplementary Material

Supplementary Figures S1-S2

Supplementary Data and Tables S1-S4

## Data Availability

All data used in the present study is available on the TCGA (https://portal.gdc.cancer.gov/), METABRIC (https://ega-archive.org/studies/EGAS00000000083) and GTEX (https://gtexportal.org/home/datasets) web portals.

## Open Access

Open access for this article was enabled by the participation of University of York in an all-inclusive *Read & Publish* pilot with Portland Press and the Biochemical Society under a transformative agreement with JISC.

## Abbreviations

BRCA: breast cancer
CEA: carcinoembryonic antigen
DEG: differentially expressed gene
FDR: false discovery rate
ML: machine learning
MRA: master regulator analysis
NES: normalized enrichment score
NGS: next-generation sequencing
OS: overall survival
TME: tumor microenvironment
VST: variance-stabilizing-transformed
