# Supplementary material for: Detection of subtype-specific breast cancer surface protein biomarkers via a novel transcriptomics approach
Source: Biosci Rep. 2021 Dec 7;41(12):BSR20212218. doi: 10.1042/BSR20212218 (PMC8655506; doi:10.1042/BSR20212218)
Supplement: Supplementary Figures S1-S2 [file BSR-2021-2218_supp.pdf]

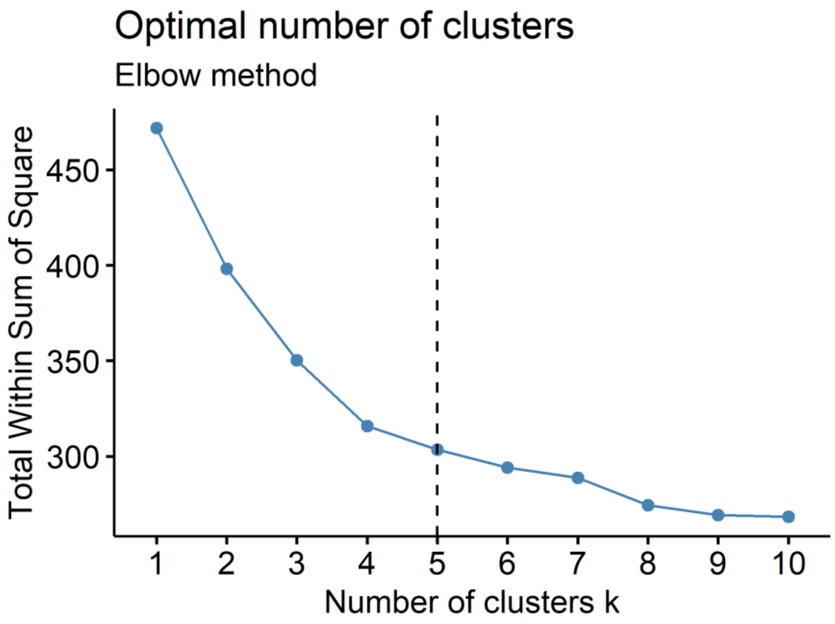

Figure S1. Elbow Plot. Optimal number of clusters was determined using the *fviz_nbclust* function from the factoextra R package (v1.0.7).

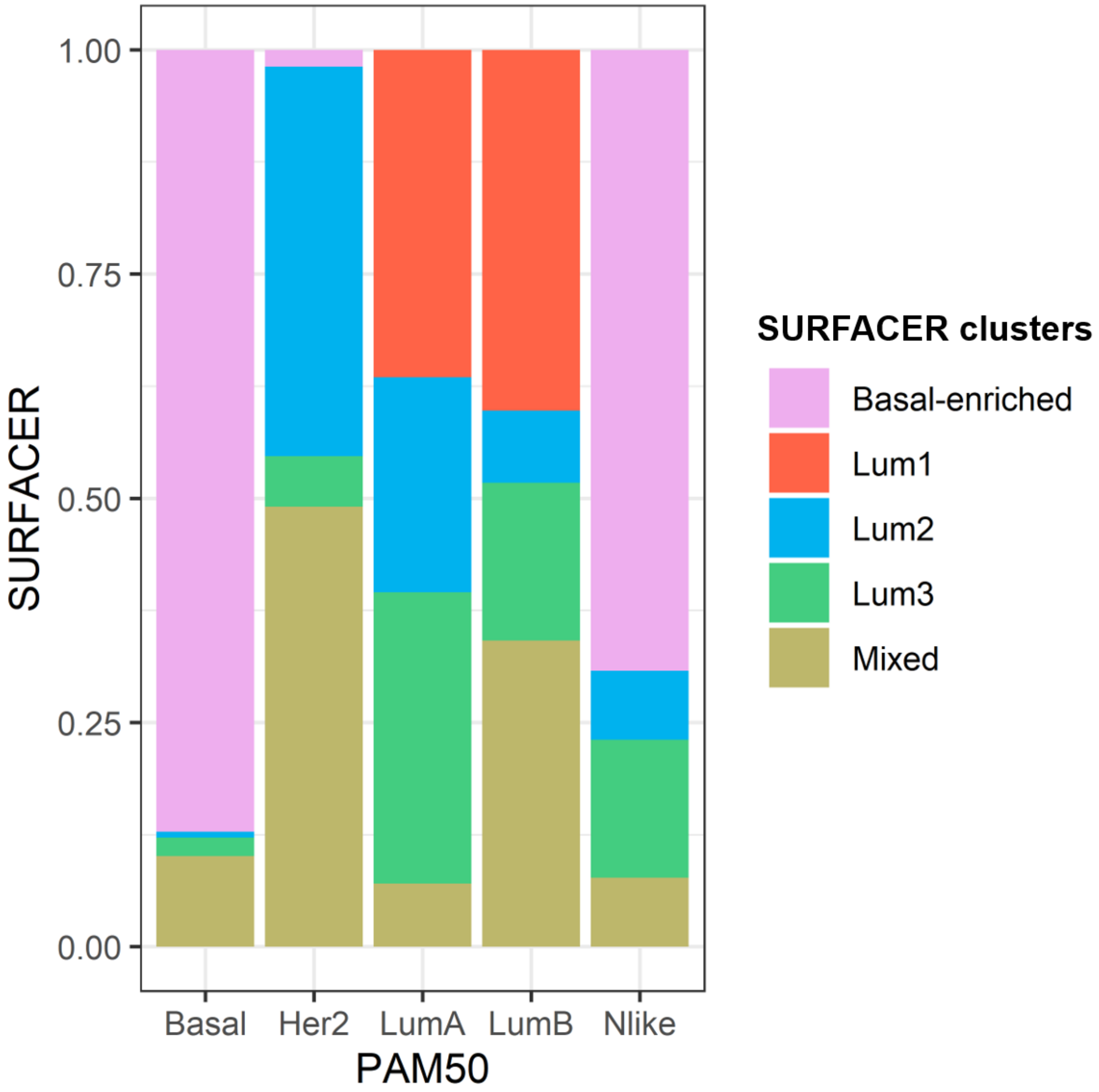

Figure S2. Proportion of SURFACER clusters in PAM50 intrinsic classification in the TCGA cohort.
